# Oriental Wisdom

**Published:** 1874-04

**Authors:** 


position from the general theory of inflammation; and the author stated that the second assumption would account for all the effects which counter-irritants are known to produce in the treatment of various diseases. A stimulant action might aggravate the disease in the first stage of inflammation, and counter irritants were known to produce this effect occasionally. At other times a stimulant action might in this stage assist the disease through its natural progress, by developing the second stage of inflammation. An instance of this effect occurred when the pain of pleurisy was relieved by a blister. In such a case the disease was not checked, but the effusion separated the pleuræ, and the pain was relieved. In the second stage of inflammation, and especially in chronic cases, a stimulant action was most likely to promote health, and it was in such cases that counter-irritants were most safely employed. A similar remark might be made with regard to cases of local debility, in the treatment of which counter-irritants were found useful. Quantitative differences were found to exist in the effects of counter-irritants according, first, to the proximity of the irritant to the seat of the disease, and, secondly, to the degree of the artificial irritation produced; and these differences were easily explicable on the supposition that the influence exerted by the counter-irritant upon the disease was of a stimulant nature.

### ORIENTAL WISDOM.

We have often heard of the "wise men of the East;" but we never appreciated the fullness of their wisdom till we read a letter on the medical doctrines of India' by Dr. Hugg, in London *Medical Times and Gazette.* He tells us that it was deemed *most unlucky* to summon a doctor away from his dinner, bed, the Church, or the theater; *most ill omened:* an extraordinary and truthful fact which ought to be impressed on the minds of modern patients. To gain the confidence of families, the physician, clean and neat, should carry an umbrella, have an agreeable voice, a small tongue, straight eyes and nose, thin lips, short teeth, and thick bushy hair, which retains its vigor; he should have a knowledge of books, and be kind to his pupils.

### A TEST FOR CARBOLIC ACID.

Plugge has observed that when a solution containing carbolic acid is mixed with a solution of the subnitrate of mercury, not only is the mercury reduced, but the liquid acquires an intense red color. Further experiments showed that the presence of traces of nitrous acid was essential to this reaction, and that it could be employed as a test for carbolic acid. He found that the color was still quite evident when only one sixty-thousandth part of carbolic acid was present. Still smaller quantities could be detected by this test if carefully applied, but the amount of nitrous acid must be very small. —*Jour. of App. Chem.*

### Miscellaneous.

### A LETTER.

CHICAGO, Feb. 16th.

TO THE EDITOR OF THE DENTAL REGISTER:

In the February No. of the REGISTER appears an article with the heading "A New Method of Pivoting," by B. Ban-

